# Impact of changes to International Commission on Radiological Protection models on occupational thorium ore dust intake

**DOI:** 10.1093/rpd/ncaf031

**Published:** 2025-04-02

**Authors:** Gregory S Hewson, Martin I Ralph, Marcus Cattani

**Affiliations:** Edith Cowan University, School of Medical and Health Sciences, 270 Joondalup Drive, Joondalup, WA 6027, Australia; Edith Cowan University, School of Medical and Health Sciences, 270 Joondalup Drive, Joondalup, WA 6027, Australia; Edith Cowan University, School of Medical and Health Sciences, 270 Joondalup Drive, Joondalup, WA 6027, Australia

## Abstract

Workers involved in mining and processing naturally occurring radioactive materials (NORMs) are potentially exposed to dust containing alpha particle emitters. The objective of this study is to summarize the key impacts of the latest International Commission on Radiological Protection (ICRP) biokinetic model for thorium ore dust intake and to identify model parameters that require further investigation. The dosimetric significance of thorium ore dust exposure has varied widely over time owing to progressive changes in the inhalation dose coefficients. These changes had a significant influence on radiation protection practices in the Western Australian mineral sand industry, including research initiatives and implementation of control measures. Estimated doses to workers exposed to NORM dust have increased because of the most recent ICRP recommendations. Consequently, we highlight the need for future research, especially in relation to appropriate model input parameters specific to the NORM exposure situation and potential studies investigating the health status of past long-term workers.

## Introduction

The Western Australian mineral sand industry has been in continuous operation for about 70 y and continues to be an important supplier of titanium and zirconium feedstocks, together with the mineral monazite, a rare earth phosphate, an important source of critical minerals essential for energy transition. External and internal radiation exposure in the industry may be significant depending on the concentration of monazite associated with the heavy mineral concentrate being processed, as monazite contains 5% to 7% thorium and 0.1% to 0.3% uranium [1]. Other heavy minerals (ilmenite, rutile, and zircon) also contain thorium and uranium, but at much lower concentrations. The mined sands are concentrated via wet gravity techniques, where the heavier minerals are separated from the lighter gangue materials. The heavy mineral concentrate is then processed in a ‘dry separation’ plant, where the individual heavy minerals are separated by physical processes that utilize the magnetic susceptibility, electrical conductivity, density, and size properties of the minerals. While external radiation from stockpiles of monazite-rich materials may be significant, the primary radiological concern in the industry in the past was the intake of monazite (and hence thorium) dust [2, 3]. Concerns about health risks to industry workers arise primarily through the potential for inhalation of thorium series radionuclides, the inherent dustiness of physical mineral separation processes, the operating duration of the industry, the number of long-term (>10 y) workers, and the relative insolubility of monazite.

Owing to their physical properties, heavy minerals are highly refractory to weathering and are essentially insoluble in biological fluids, including lung fluid [4]. Consequently, monazite dust deposits in the lower lung are ardently retained, resulting in long-term alpha particle irradiation of lung tissues.

Over 25 y ago, individual monitoring of worker intake via several bioassay research studies (i.e. *in vitro* and *in vivo* tests) was undertaken in the Western Australian mineral sand industry [1]. The results at that time indicated that several long-term (>10 y) workers received average annual effective doses from inhalation of thorium ore dust greater than the current occupational dose limit of 20 mSv over their employment period [1]. Similar conclusions may be drawn from bioassay investigations undertaken at other industrial operations involving thorium. Terry and Hewson [5] summarized literature values of *in vivo* thorium lung burdens and thoron-in-breath measurements and the reported values (at that time) were of the same order as those found in the Western Australian studies. These studies highlighted that further research was needed into the assumptions inherent in the metabolic and dosimetric models for thorium recommended by the International Commission on Radiological Protection (ICRP), especially for the intake of dusts where monazite or other thorium-rich dusts are processed.

Prior to the latest ICRP biokinetic models [6, 7], researchers questioned the appropriateness of the previous models and the uncertainties arising from the recommended default parameters [8–11]. A recent study summarizes and analyses past radiation research in the mineral sand industry and discusses the implications of revised biokinetic models [12]. These studies highlighted the importance of seeking material-specific data for model parameters such as aerosol particle size and lung and gut solubilities rather than simply adopting the ICRP default values, especially if doses were assessed as being significant. The bioassay studies on workers exposed to inhaled thorium minerals consistently showed lower thorium excretion in urine [13] than predicted by the earlier models and longer retention of thorium dust in the lung [5]. These results indicate less thorium absorption into the blood after lung deposition for Type S (low solubility) materials.

The updated models recommended in the ICRP’s series of publications on the occupational intake of radionuclides (OIRs) [6, 7] provide an opportunity to review the impact of changes to thorium dosimetry and to assess potential improvements to industry radiation protection practices, such as protocols for estimating intake of thorium dust and the subsequent dose.

The relevant ICRP models applied to the derivation of the inhalation dose coefficients used in the Western Australian mineral sand industry over the last 40 y are listed in Table 1. The Western Australian regulatory agency responsible for radiation protection in mining operations (now the WorkSafe Mine Safety Directorate) subsequently adopted (indirectly) the ICRP models by specifying, in government guidelines, the dose coefficients to be applied to intakes of naturally occurring radioactive material (NORM) dust. The latest iteration of the guideline [14] is based on the work of Ralph et al. [15], which adopts the latest ICRP dose coefficients as per the ICRP Dose Viewer application [16].

**Table 1:** ICRP biokinetic models relating to assessment of inhaled radioactivity and applying over the life of Western Australian mineral sand industry operations.

Publication	Year	Model^a^
ICRP 2	1960	Respiratory tract model
ICRP 30	1979	Task group lung model (TGLM)
ICRP 66	1993	Human respiratory tract model (HRTM)
ICRP 69	1995	Thorium biokinetic model
ICRP 130	2015	Revised human respiratory tract model
ICRP 137	2017	Thorium biokinetic model

^a^Excluding gastrointestinal and human alimentary tract models since absorption of inhaled thorium dust in the alimentary tract is an insignificant contributor to dose.

An excellent overview of the various ICRP methods and models used for assessing and interpreting internal doses (as listed in Table 1) is provided by Paquet et al. [17]. The authors discuss the various changes to the models, in particular the changes arising from the ICRP’s revised human respiratory tract model (HRTM) [6] and highlight the uncertainties in model parameters and inter-worker variability which impact interpretation of internal dose.

## Methodology

The objectives of this study were to review the impact of progressive refinements on ICRP biokinetic models for chronic thorium inhalation on dose assessments and to investigate alternate model parameters to refine dose estimates for workers occupationally exposed to thorium ore dust.

The inhalation dose conversion factors for thorium ore dust applied to the Western Australian mineral sand industry over time were reviewed to document the extent of changes and the potential impact on previously reported doses. To understand the key model assumptions resulting in dose coefficient changes, applicable ICRP biokinetic models [6, 18–20] and relevant literature were assessed via a literature search using search engines such as PubMed, Google Scholar, and Scopus. The search criteria were selected using keywords and inclusion and exclusion criteria to identify articles mainly relating to thorium dust intake as encountered in industries involved in mining and processing NORM, with a focus on mineral sand and monazite dust.

The ICRP Dose Viewer software application [16] was used to extract inhalation dose coefficients for thorium-series radionuclides as a function of particle size and lung solubility type. In this study, the least soluble form (Type S) of the radionuclides was selected, reflecting the relative insolubility of mineral sand dust. The Dose Viewer and ICRP Publication 71 [21] were used to investigate the changes in tissue dose equivalents over time. Taurus, a commercially available internal dosimetry software [22], was applied to the latest ICRP models to assess the impact of alternative aerosol parameters likely to be encountered in the Western Australian mineral sand industry on the inhalation dose coefficients for thorium series radionuclides. The relative impact was obtained by contrasting the revised parameter value with the ICRP OIR series default values. Chronic intake of Type S ^232^Th was selected as the exposure scenario and the parameters investigated with the Taurus software included aerosol particle physical characteristics (activity median aerodynamic diameter [AMAD], geometric standard deviation [GSD], shape, and density), aerosol particle absorption, breathing rates, and activity levels. The outputs from the software were downloaded to Microsoft Excel to consolidate and analyse the data.

## Results

Other authors have commented on the impact of thorium dosimetry changes following previous refinements to the ICRP biokinetic models. Leggett [23] discusses the substantial differences between the ICRP 30 and ICRP 66/69 models, particularly the changes to tissues doses and the significant changes to predicted thorium excretion rates and hence implications for bioassay. Leggett highlights that the ICRP 69 model ‘…*predicts much longer retention of thorium in the skeleton and other soft tissues, and consequently much longer retention in the body*’. In relation to chronic intakes it was noted that daily urinary excretion was approximately five-fold lower than predicted by ICRP 30. Phipps et al. [24] investigated the impact of the revised ICRP 66 respiratory tract model, the ICRP 69 biokinetic model for thorium, and the changes in tissue weighting factors in ICRP 60 to thorium dose coefficients. The authors found a substantial rise (up to a factor of 20) in the annual limit on intake for Type S ^232^Th compared with the previous ICRP 30 model. These authors also highlighted that the model changes improve the feasibility of urine bioassay for significant intakes, which had not been the case with the ICRP 30 model. Stehney [25] reported good agreement with the ICRP 66/69 models and the results of autopsy samples of lungs and other organs of former thorium refinery workers. The agreement was attributed to the new models ascribing longer retention times for thorium in the lungs and in the liver compared to the ICRP 30 model. Jaiswal et al. [26] compared measured and computed total body content and daily urinary excretion of thorium in five occupationally exposed workers. These authors found relatively good agreement between the measured values and those computed from the ICRP 66/69 models and concluded that these models are expected to yield reasonably accurate estimates when applied to bioassay measurements.

No recent studies were found analysing the impact of the most recent ICRP models for thorium (ICRP 130/137) and implications for thorium dosimetry and bioassay.

### Inhalation dose coefficients

The significance of thorium ore dust exposure has varied by factors in excess of three (up and down) as progressive changes were made to inhalation dose coefficients derived from ICRP biokinetic and dosimetric models. The inhalation dose coefficients derived from various ICRP publications for key thorium-series radionuclides are summarized in Table 2. The inhalation dose coefficient (DC_inh_) listed under ‘Th series’ shows the overall impact of the progressive changes to ICRP biokinetic models. Table 2 illustrates that significant changes have occurred since ICRP Publication 2 [27] as biokinetic models have become more sophisticated, reflecting an improved understanding of the retention and excretion of inhaled radionuclides. For example, DC_inh_ (in Sv.Bq^−1^) for a 5 μm AMAD, Type S ^232^Th aerosol was listed as 13 × 10^−5^ in ICRP 30, 1.2 × 10^−5^ in ICRP 68 (11-fold reduction), and 5.4 × 10^−5^ in ICRP 137 (4.5-fold increase). Clearly, these changes have substantial impact on dose assessment protocols following thorium intake and the significance of past reported doses.

**Table 2:** Changes in ICRP inhalation dose coefficient (DC_inh_) for ^232^Th series radionuclides.

Publication	DC_inh_ (Sv.Bq^−1^) for Type S aerosol (unless indicated otherwise)				
	^232^Th	^228^Th	^228^Ra	^224^Ra	Th-series
ICRP 2^a^	1.9E-5	1.9E-5	0.8E-5 (sol)	0.01E-5 (sol)	4.6E-5
ICRP 30^b^ (5 μm AMAD)	13E-5 (Y)	3.6E-5 (Y)	0.05E-5 (W)	0.03E-5 (W)	17E-5
ICRP 68 (5 μm AMAD)	1.2E-5	2.5E-5^c^	0.17E-5 (M)	0.24E-5 (M)	4.1E-5
ICRP 137 (5 μm AMAD)	5.4E-5	2.3E-5	2.2E-5	0.11E-5	10E-5
ICRP 137 (10 μm AMAD)^d^	2.6E-5	1.4E-5	1.3E-5	0.065E-5	5.4E-5
Ratio 137:30^e^	0.42	0.92	44	3.7	0.59
Ratio 137:68^e^	4.5	0.64	13	0.46	2.4

^a^ICRP 2 contains provisional values for ^232^Th and Th-nat. These were expressed as the MPCs in air based on a 40-h week. The MPCs were converted to DC_inh_ by dividing the yearly intake (MPC × 2400 m^3^) into 0.05 Sv. Radium figure based on ‘soluble’ form.

^b^ICRP 30 published DC_inh_ for 1 μm AMAD particles, which is related to an annual dose limit of 0.05 Sv. A particle size correction formula was used to determine the DC_inh_ for the other sizes. The Western Australian mineral sand industry uses a default AMAD of 5 μm, as approved by the regulatory authority. A much greater difference was evident when 1 μm DC_inh_ was used.

^c^ICRP 119 provided a corrected value for ^228^Th based on improved biokinetic modelling of radioactive progeny formed in body organs. This reduced the ICRP 68 dose coefficient for the thorium series from 4.8E-5 to 4.1E-5.

^d^DC_inh_ values shown for AMAD of 10 μm since past reported particle size measurements from personal cascade impactors worn by mineral sand workers showed typical values of 10 μm or more with a GSD of 3.0 [28, 29].

^e^The ratios refer to ICRP 137 DC_inh_ to ICRP 30 DC_inh_ and ICRP 68 DC_inh_ for 5 μm AMAD aerosols.

**Figure 1:**
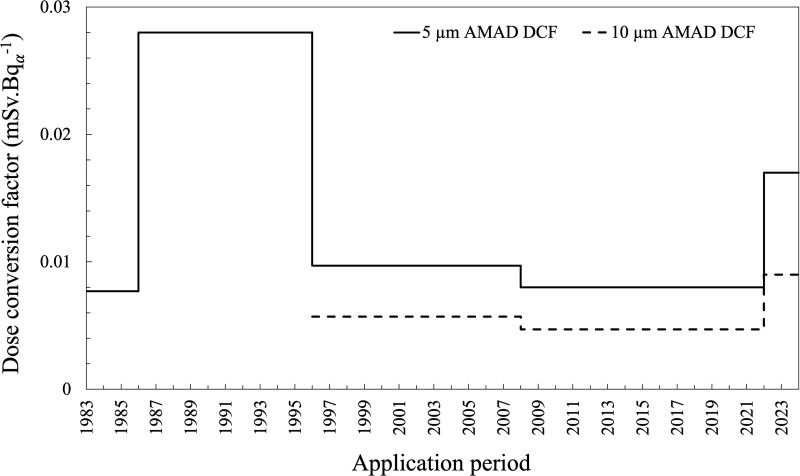
Inhalation DCFs applying to thorium ore dust intake in the Western Australian mineral sand industry, expressed as mSv.Bq_α_^−1^.

Workplace airborne radioactivity concentrations in the Western Australian mineral sand industry are typically measured as total long-lived alpha activity concentration (Bq_α_.m^−3^) and the alpha activity intake is calculated using the exposure time (typically hours worked per year) and an assumed breathing rate (in m^3^.h^−1^). The factor to convert alpha activity intake to dose has been referred to as the dose conversion factor (DCF_inh_) [14]. Figure 1 shows the DCF_inh_ values (expressed as mSv per long-lived alpha activity of the thorium series) that have been applied to the Western Australian mineral sand industry since 1983 [3]. DCF_inh_ for 5 μm AMAD and 10 μm AMAD aerosols are shown as some sites had regulatory approval to apply a 10 μm AMAD DCF_inh_ for some work categories on the basis of an AMAD measurement programme. The DCF_inh_ for 2021 onwards was derived from the ICRP Dose Viewer [16], using the same aerosol parameters as described earlier and listed later in Table 5. Formal recording and reporting of internal doses only commenced in 1983, although the records between 1983 and 1985 were based on a much smaller and incomplete database [2].

**Figure 2:**
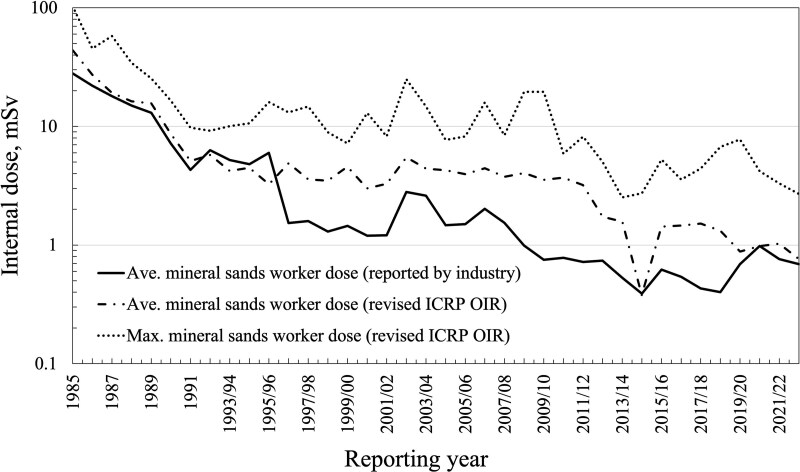
Reported (by industry) and revised estimated internal doses to Western Australian mineral sand workers. Revised as per the ICRP dose coefficients listed in the OIR data viewer [15].

The impact of the latest change to the Th-series DCF_inh_ on reported doses in the Western Australian mineral sand industry can be estimated by applying the Th-series ratios listed in Table 2 to the internal doses derived from airborne radioactivity concentrations reported by the industry over a period of interest. Ralph [30] provides a tabulation of mean and maximum internal doses and total long-lived alpha activity concentrations in air and mean working hours as reported by the mineral sand and other NORM-related industries from 1977 to 2022. Figure 2 shows the output of this analysis for the period 1985 to 2022. The line designated ‘Ave. internal dose (revised ICRP OIR)’ is the trend based on applying the same DCF_inh_ to the mean annual intake over each exposure year. The revised dose estimate is likely conservative as the analysis applied the default 5 μm AMAD DCF_inh_ across all sites and work categories. Intakes derived from past bioassay measurements on workers will produce different dose estimates using the new models, and further studies are underway to understand the implications.

### Review of model assumptions for inhaled thorium ore dust

A revised lung model (referred to as the HRTM) was introduced in 1994—ICRP 66 [19] to replace the earlier ICRP 30 lung model [18]. The HRTM changed the deposition fractions of inhaled aerosols as a function of AMAD. Lower deposition in the lung for coarser aerosols led to lower uptake to the blood and consequently lower doses to bone tissues. There has also been a change in the dosimetry of alpha particles, including less absorption of alpha energy emitted by radiosensitive cells in the airways [24].

The revised biokinetic model for thorium in ICRP 69 [20] was aligned with the model for actinides and resulted in the natural thorium radionuclides delivering less dose to bone surfaces as they became buried in bone volume. This was a significant contrast to ICRP 30, which allowed the thorium to irradiate bone surfaces with a removal half-life of 50 y.

ICRP 137 [7], which contains the biokinetic model for thorium, resulted in further revisions while the revised HRTM [6] provided greater retention in the alveolar interstitium (AI) region for insoluble particles (i.e. monazite dust), and approximately one-third of the alveolar deposits were sequestered in the interstitium. This increased the lung dose per intake for long-lived alpha emitters, such as thorium. The HRTM revisions were based on further human studies that consistently showed long-term retention in the alveolar interstitial region.

A comparison of the key changes to thorium series inhalation dose coefficients to organs and tissues between ICRP 68 and ICRP 137 was facilitated by examining the equivalent dose coefficients published in ICRP 71 [21] and those available via the electronic annex provided as a supplement to the ICRP 130 OIR series [16]. Table 3 illustrates the extent of the changes in the various tissues, and it is evident that the modifications to the thorium biokinetic model led to a significant reassessment of tissue doses following the intake of insoluble thorium compounds.

**Table 3:** Changes in equivalent dose coefficients following inhalation of Type S thorium series radionuclides.

Tissue	Effective dose contribution ICRP 137 (^232^Th) (%)	Equivalent dose ratio, ICRP 137:ICRP 71^a^		
		^232^Th	^228^Th	^228^Ra
Lungs	72.7	3.4	0.73	1.9
Lymphatic nodes^b^	21.3	–	–	–
Red marrow	1.7	1.1	0.61	0.82
Extra-thoracic airways	1.4	2.7	2.8	2.7
Liver	0.53	2.4	0.98	1.4
Bone surfaces	1.1	0.34	0.30	0.43
Kidneys	0.17	3.5	2.54	3.5

^a^Based on 1 μm AMAD aerosols of Type S solubility.

^b^Lymphatic nodes were not separately listed in past ICRP publications.

The revised HRTM in the ICRP 130 also introduced updated absorption parameters for inhaled and ingested thorium. The recommended values are listed in Table 4. The changes in *f*_r_ and *s*_r_ between ICRP 66 and ICRP 130 resulted in a reduction in rapid absorption in the extra-thoracic airways and an increase in the lungs [6]. Absorption from the alimentary tract (*f*_a_) was reduced by two orders of magnitude for thorium cleared from the respiratory tract compared with thorium directly ingested (i.e. via diet).

**Table 4:** Changes in default absorption parameter values for inhaled and ingested thorium.

Absorption parameter	ICRP 66/69 Type S (slow)	ICRP 130/137^a^ Type S (slow)
*f*_r_—fraction dissolved rapidly	0.1%	1%
*s*_r_—rapid dissolution rate (d^−1^)	100	3
*s*_s_—slow dissolution rate (d^−1^)	1 × 10^–4^	1 × 10^−4^
*f*_a_—absorption from alimentary tract^b^	5 × 10^–4^	5 × 10^−6^
*f*_a_—ingested materials—all forms	–	5 × 10^−4^

^a^ICRP 130 [6] highlights that the GSD associated with experimental data for these values is very large [9–14] and therefore, there are large uncertainties in the rounded numbers shown in the table.

^b^For inhaled material deposited in the respiratory tract and subsequently cleared by particle transport to the alimentary tract.

### Aerosol and intake assumptions

Taurus internal dosimetry software [22] was used to investigate the impact of applying alternate aerosol parameters more aligned to monazite dust intake. ICRP 130 default assumptions for insoluble mineral dust, as encountered in mineral sand industries, are tabulated in Table 5, together with values considered more appropriate for the intake of thorium-bearing dust as encountered in Western Australian mineral separation plants. An alternative dynamic shape factor of 1.25 was selected considering the formation of monazite sand grains after protracted wind and wave action (refer footnote to Table 5). Hence, the aerodynamic behaviour of monazite dust should be closer to that of spheres (shape factor of 1). The use of alternate values can significantly alter the assessed dose, confirming the importance of understanding the actual particle size distribution (AMAD and GSD) and lung solubility (particle dissolution rate) of inhaled particles.

**Table 5:** Aerosol characteristics: ICRP model default parameters vs. monazite dust.

Aerosol parameter	ICRP default	Monazite dust	Effect^a^
Thorium series solubility type	S	S	–
AMAD (μm)—personal air sampling^b^	5	>10	0.53
Aerosol distribution shape GSD	2.5	3.0	1.4
Shape factor^c^, *χ*	1.5	1.25	–
Mass density, *ρ* (kg.m^−3^)	3.0	5.2	–
*χ* */**ρ*^d^	0.5	0.24	1
Lung solubility (*s*_s_)	1 × 10^−4^	3.5 × 10^−5^	1.7

^a^Factor to be applied to dose coefficient compared to ICRP default value. Derived using Taurus internal dosimetry software [22].

^b^Personal cascade impactor measurements on mineral sand plant workers typically show AMADs of 10 μm or more with a GSD of 3.0 [29, 28].

^c^Spherical particles have a dynamic shape factor of 1.0, while a shape factor of 1.5 is characteristic of plate-shaped particles. Quartz dust is reported to have a shape factor of 1.36 [31]. Monazite dust is typically cuboid-shaped, similar to other mineral dust, and such particles are characterized by a shape factor of ~1.25 [32].

^d^Total and regional deposition fractions in the human respiratory tract generally decrease with increasing *χ*/*ρ* and a larger decrease is observed with a smaller AMAD [33].

### Mode and rate of breathing

ICRP 130 [6] uses a breathing rate for workers engaged in ‘light’ activity (31% rest at 0.54 m^3^.h^−1^ and 69% light exercise at 1.5 m^3^.h^−1^). ‘Heavy’ exercise is allocated a breathing rate of 3 m^3^.h^−1^, and an overall breathing rate of 1.5 m^3^.h^−1^ is likely more appropriate for the more strenuous work activities typically encountered at mining and mineral processing sites (i.e. 20% rest, 65% light work, 15% heavy work). ICRP 89 [34] indicated an even higher average breathing rate of 1.7 m^3^.h^−1^ for males conducting heavy work, such as construction workers.

Table 6 summarizes the impact of modifying ICRP’s default breathing parameters to reflect the likely rate and mode of breathing by workers involved in the operation and maintenance of mineral sand plants or other industrial activities involving NORM.

**Table 6:** Influence of breathing rate and mode of breathing.

Breathing parameter	ICRP default	Alternative	Effect^a^
Rate, light vs. moderate activity^b^, m^3^.h^−1^	1.2	1.5	1.2
Rate, light vs. heavy activity^b^, m^3^.h^−1^	1.2	1.7	1.4
Mode of breathing^c^	Nose	Mouth	2.6
Mode of breathing^c^	Nose	0.85 N/0.15 M	1.4

^a^Factor to be applied to intake or dose coefficient compared to default value.

^b^The adjustment factor for breathing rate is applied to the intake (exposure) obtained from airborne radioactivity sampling and exposure time records.

^c^Inhalation dose coefficient calculated using the Taurus internal dosimetry software application based on chronic inhalation of 10 μm AMAD, Type S aerosol, and heavy work.

## Discussion

### Impact of changes to inhalation dose coefficients

There have been relatively few publications of research into thorium biokinetics and dosimetry since the early 2000s, especially in relation to better defining material-specific data for aerosol characteristics and absorption parameters for workers exposed to NORM dusts. The need to revisit past exposure and bioassay data for mineral sand and other NORM workers is indicated by the 2.4-fold increase in the inhalation dose coefficient for the thorium series. Bioassay studies may now be able to determine doses from chronic intakes that are significantly below the annual limit. A recent study discusses the substantial differences in the predicted urinary thorium excretion between the ICRP 30 and ICRP 130 models for chronic intakes of Type S ^232^Th [12]. An order of magnitude difference in predicted daily thorium excretion was noted, and this has significant implication for interpretation of bioassay results and estimation of intake.

Some key points arising from Table 2 include the following (based on comparisons between the DC_inh_ for 5 μm AMAD aerosols).

1. ^232^Th DC_inh_ decreased 10-fold between ICRP 30 and 68 and then increased by more than four-fold in the more recent ICRP 137.
2. ^228^Th DC_inh_ has not varied greatly over the three ICRP publications; however, it was the primary contributor to the ^232^Th series effective dose coefficient in ICRP 68.
3. ^228^Ra DC_inh_ increased more than three-fold between ICRP 30 and ICRP 68, and then increased 13-fold in ICRP 137 (a 44-fold increase from ICRP 30). For ^228^Ra, the increase is related to the change in solubility class from Class W/Type M to Type S. This is because of the assumption that ^232^Th series progeny in the Class S material stays with the parent and has similar solubility to the particle matrix.
4. ^224^Ra DC_inh_ has gone up and then back down. It does not contribute significantly to the thorium series effective dose coefficient. Similar to ^228^Ra, the solubility class was changed to reflect the particle matrix.

ICRP 30 recommended that the default AMAD was 1 μm for worker intake, which was originally applied in the Western Australian mineral sand industry during the 1980s and the early 1990s. Subsequent regulatory approval for a change to a default AMAD of 5 μm reduced the ICRP 30 DC_inh_ for ^232^Th from 31 × 10^−5^ to 13 × 10^−5^ Sv.Bq^−1^, a 2.4-fold reduction.

The tissue weighting factor (*W*_T_) for bone surfaces decreased by a factor of three in ICRP 60 [35], from 0.03 to 0.01. This was important for thorium, as bone surfaces were the major contributor to the committed effective dose in ICRP 30. As a consequence of the definition of effective dose changing over time, comparison of doses over different time periods is problematic, other than from a regulatory compliance perspective. In relation to doses derived from personal air sampling measurements, the estimated intakes (e.g. Bq.y^−1^) for workers are independent of the models and can be used with an updated DC_inh_ to reassess the dose.

From a radiation protection perspective, the changes documented in Table 2 had a significant impact on the operational practices of the Western Australian mineral sand industry. A similar impact would likely have been experienced by industries elsewhere involved in mineral sand, rare earth, and NORM processing, where thorium dust was encountered. The ICRP 30 change triggered a concerted research effort into thorium retention and excretion and prompted the industry to invest in large-scale capital work to improve dust control and ventilation in separation plants [36].

Figure 1 illustrates a particular conundrum for industry, given the magnitude of change in each application period and the corresponding impact on the estimated internal doses to industry workers. The average assessed annual effective dose to mineral sand workers, from the same intake, increased substantially in the mid-1980s, decreased substantially in the mid-1990s and finally increased again in the early 2020s. For example, gross alpha activity intake of 500 Bq_α_.y^−1^ of 5 μm AMAD thorium-bearing dust corresponded to an annual effective dose of 3.8 mSv prior to 1986 (c.f. then dose limit of 50 mSv), 14 mSv between 1986 and 1995, 3.4 mSv between 1996 and 2001, and 8.3 mSv since 2021. The latest change resulted in a two-fold increase in the derived DCF_inh_ for the thorium series assuming a 5 μm AMAD, Type S aerosol.

From a historical perspective, it is important to recognize that radiation protection practices in the industry, such as the level of monitoring, research effort, and implementation of control measures, were related to the perceived level of hazard at the time. In contrast to the substantial investment in research and engineering in the early to the mid-1990s, the late-1990s saw a cessation of research effort and less emphasis on ALARA measures as internal doses were reduced by four-fold due to ICRP 68 changes to dose coefficients (as per Fig. 1). For 25 y (1996–2021), the industry has considered itself to have internal radiation doses well under control (Fig. 2). However, the latest biokinetic model changes, as per ICRP 130 and ICRP 137, result in higher doses (from the same inhalation intake) and require a renewed focus on assumptions used in the dose assessment methodology and possibly additional as low as reasonably achievable (ALARA) measures and changes to operational practices. Figure 2 illustrates that maximum internal doses from intake of radioactive dust in the Western Australian mineral sand industry can still be a significant fraction of the 20 mSv annual dose limit and that doses above 10 mSv.y^−1^ were being recorded up to 2010. The revised exposure data indicates that bioassay is likely to be feasible for workers with several years (e.g. >5 y) of chronic intake [12]. A urinary thorium excretion rate for workers of 1 ng.l^−1^ (4 μBq.l^−1^) or more is able to be detected using inductively coupled plasma mass spectrometry (ICP-MS); however, it will be important to distinguish occupational intake from environmental intake of NORM [12]. This finding may also be applicable to workers in other NORM industries where radioactive dust intake is of a similar order over several years.

### Changes in equivalent dose coefficients

Table 3 highlights that for ^232^Th, the tissue equivalent dose per intake was reduced to bone surfaces (three-fold) but increased by approximately three-fold in the lungs, kidneys, and extra-thoracic airways. The lymphatic nodes were assigned a much higher equivalent dose per intake. This tissue, which has not been separately listed in the past, is now a significant contributor (21%) to the effective dose coefficient. The overall impact of the updated biokinetic and HRTM models was a 4.5-fold increase in the effective dose coefficient of ^232^Th compared to that of ICRP 68/71 (Table 2).

For ^228^Th, the key changes between ICRP 68/71 and ICRP 137 were increased dose per intake to the extra-thoracic airways and slightly lower dose per intake to the lungs, with an overall reduction in the effective dose coefficient. For ^228^Ra, the effective dose coefficient increased two-fold, primarily by assigning Type S solubility to radium and, consequently, increasing the equivalent dose per intake to the lung and extra-thoracic airways and reducing the dose per intake to bone surfaces and red bone marrow.

While there has been a significant change in the equivalent dose per intake to the kidneys for ^232^Th, ^228^Th, and ^228^Ra, this tissue is a minor contributor (<<1%) to the overall effective dose from Type S thorium series radionuclides. The lungs now contribute 73% of the effective dose following inhalation of Type S thorium.

Overall, the changes in tissue dose equivalents summarized in Table 3 indicate that health surveillance programmmes in the mineral sand industry and other NORM industries where thorium ore dust may be encountered should focus on monitoring respiratory symptoms and lung function following chronic long-term intake. These changes also have implications for bioassays in NORM industries in that lung retention of thorium is increased and urinary thorium excretion is decreased. This could improve the feasibility of thoron-in-breath measurement. However, improved detection levels for thorium in urine may be necessary, and urine bioassays of workers may be more problematic in areas of high natural background where ingestion of thorium via diet could complicate the interpretation of results.

### Impact of aerosol parameter assumptions

The updated thorium model in ICRP 137 does not assign material-specific absorption parameters to Type S thorium materials but instead assigns the default values as per Table 4. However, in reviewing experimental data for thorium dioxide, the ICRP [7] highlights that absorption could be even lower than that assumed for default Type S. Autopsy studies of thorium ore and refinery dusts show large amounts of ^232^Th remaining in the lungs 6–30 y after the end of employment [37]. This result accords with bioassay measurements on a Western Australian mineral sand industry worker whose thorium lung burden remained unchanged during 4 y of follow-up post-cessation of thorium dust intake [38]. A biological clearance half-time from the alveoli of 20,000 d (3.5 × 10^−5^ d^−1^) was inferred based on this finding. Hence, it seems reasonable to assume that thorium inhaled via a mineral sand dust matrix will likely have lower absorption parameters than those indicated in Table 4. The order of magnitude differences can be contemplated given the footnote to Table 4 (i.e. the experimental data have very large GSDs).

In relation to the effective dose via the thorium cleared from the extra-thoracic region to the alimentary tract, it is noted that *f*_a_ is very low (Table 4); hence, the internal dose arising from the ingestion pathway for Western Australian mineral sand industry workers and likely other NORM workers is considered insignificant.

In relation to the aerosol and intake assumptions (Table 5), the particle size of thorium dust is especially important if it varies significantly from the assumed default AMAD of 5 μm. Previous measurements in the Western Australian mineral sand industry indicated an AMAD of 10 μm or more based on personal cascade impactor tests [28]. However, the Western Australian particle size measurements were undertaken more than 30 y ago, and only one study has been reported in similar type operations since that time [39]. Positional impactor measurements at two Indian mineral sand plants showed AMADs ranging from 2.7 to 15 μm with a mean of 7 μm.

In the case of coarse mineral sand or NORM dust, the shape factor and density have a negligible impact at an AMAD of 5 μm and larger. The impact is greatest for smaller aerosols (e.g. 1 μm or less), as may be encountered following the crushing, grinding, and chemical treatment of NORM minerals. Hence, these aerosol parameters may be more important during the extraction of rare earths from the monazite ores and during the production of zircon flour.

In this analysis, the slow dissolution rate (*s*_s_) for monazite dust has been allocated a value of 3.5 × 10^−5^ d^−1^ (clearance half-time of 20,000 d) based on results from thoron-in-breath measurements [38] and *in vitro* lung solubility tests [4]. This parameter affects the predicted daily urinary excretion of thorium, as protracted retention in the lung tissue results in lower excretion. Further, *in vivo* and *in vitro* bioassay studies may assist in better defining lung solubility.

### Impact of rate and mode of breathing

Western Australian mineral sand industry plants are multi-level design, dry, and hot because of the nature of the mineral separation and concentration processes. Hence, worker breathing rates are likely to be higher than the ICRP assumed rate of 1.2 m^3^.h^−1^, especially while doing manual tasks and accessing stairwells during 6 mo of warmer weather.

ICRP 130 assumes that the default mode of breathing for most workers is through the nose. However, a worker breathes partly through the nose and partly through the mouth during heavy exercise. Mouth breathing changes aerosol deposition in the respiratory tract, and there is no deposition in the anterior nasal passage and increased deposition in the lower regions of the lung, with a consequent increase in dose.

Owing to the strenuous nature of some work activities in mineral sand plants and mining operations in general, and the likely higher breathing rates, it is assumed that some level of mouth breathing will occur during daily work. In previous Western Australian mineral sand industry bioassay work [5, 40], the assumption was made of 85% nose breathing and 15% mouth breathing based on advice from an occupational physician.

In relation to the mode of breathing, the biggest impact is on workers who are predominantly mouth breathers, as their effective dose from intake of thorium dust could be nearly three times higher than that estimated using the default values. The assumption of heavy activity may be more realistic for Western Australian mineral sand industry workers, in which case their doses could be 40% higher than currently estimated. For nose breathers under the ‘heavy activity’ scenario, the ICRP allocated 94% to nose breathing and 6% to mouth breathing. Using the Taurus software, it was noted that varying the breathing rate does not have a material impact on the inhalation dose coefficient. However, the value assigned to the breathing rate has a significant impact on the exposure estimate (i.e. mean alpha activity intake) determined from personal air sampling measurements.

The above discussion indicates that the use of alternative breathing parameters may increase doses by approximately two-fold if workers are predominantly involved in heavy activity. The treatment of a small percentage of workers who are predominantly mouth breathers is problematic because the mode of breathing is not typically a physiological characteristic recorded in employee records. However, it would be appropriate to collect such information during individual monitoring (e.g. bioassay tests) or if the estimated intakes were above predetermined action levels. This will enhance the accuracy of dose assessments and ensure compliance with dose limits, even in cases where reference assumptions may lead to underestimation.

### Summary of overall impacts of changes to model parameters

Analysis of the various factors described above leads to an overall impact on committed effective doses to Western Australian mineral sand industry workers from the intake of thorium ore dust of a 6.2-fold increase compared to doses estimated using previous ICRP 68 protocols. This comprised a 2.4-fold increase in the default inhalation dose coefficient (Table 2), a 1.3-fold increase due to the adoption of alternative aerosol characteristics (Table 5), and a two-fold increase due to the use of more realistic breathing parameters (Table 6). While the alternative parameters used in this study were considered realistic, it is emphasized that they may still have large uncertainties, especially the value assigned to the slow dissolution rate (*s*_s_) and the aerosol distribution shape GSD. This analysis is based on the typical exposure situation encountered in Western Australian mineral sand operations. The impact on other operations involved in processing ores containing NORM may be different and will be dependent on the assumed aerosol characteristics and the nature of worker activities.

The evolving understanding of thorium metabolism following intake requires that regulatory protocols and guidelines for assessment of internal dose be updated to reflect current knowledge. Retrospective re-evaluation of past doses will be important to improve understanding of past health risks and to inform any future health effects study of mineral sand and other workers exposed to NORM dusts. The progressive refinements to biokinetic and dosimetric models have increased the long-term retention of thorium in the lungs with consequent long-term irradiation of lung tissue. This could lead to respiratory disorders that may not become apparent for decades and implies that suitable health surveillance programmes should be implemented for NORM industry workers.

## Conclusion

This study has shown that progressive changes in biokinetic and dosimetric models have had a substantial impact on the significance of thorium dust exposure, particularly in the mineral sand industry. The revised inhalation dose coefficients from ICRP models over time have altered worker dose estimates by large factors, affecting occupational hygiene practices, operational strategies, and research efforts, including the feasibility of bioassay studies. Our findings illustrate the need for model inputs that reflect the actual exposure scenario, including particle size, lung solubility, and activity levels, which influence dose estimates. Given the increased effective dose estimates arising from the latest models, industry-specific control measures may require reassessment to ensure effective radiation protection. Future research should focus on refining model parameters and validating dose estimates using empirical data to enhance the accuracy and reliability of thorium risk assessments in industries involved in mining and processing NORM. Regulatory agencies and industries should consider developing protocols to reassess past dose assessments to identify the changes between past estimates and current knowledge. This will be important for future health effect studies of workers and determining the need for additional health surveillance due to the latent effects of thorium retention in the lung.
